# Effects of training with flow restriction on the exercise pressor reflex

**DOI:** 10.1007/s00421-018-3911-2

**Published:** 2018-06-27

**Authors:** Patrik Sundblad, Roger Kölegård, Eric Rullman, Thomas Gustafsson

**Affiliations:** 1 Department of Laboratory Medicine, Clinical Physiology, Karolinska Institutet, SE-141 86 Stockholm, Sweden; 2 Department of Clinical Physiology, Karolinska University Hospital, SE-141 86 Stockholm, Sweden; 3 Department of Environmental Physiology, School of Technology and Health, KTH Royal Institute of Technology, Berzelius väg 13, 171 65 Solna, Sweden

**Keywords:** Exercise, Arterial pressure, Flow restriction, Pressor reflex

## Abstract

**Purpose:**

We hypothesized that 5 weeks of endurance training with blood flow restriction (R-training), providing relative ischemia and stimulation of the muscle chemoreflex, would decrease the exercise pressor reflex (EPR) when compared to training with the same workload in a free-flow condition (NR-training).

**Methods:**

10 subjects performed one-leg knee-extension training four times a week during a 5-week period. Both legs were trained with identical workload, with one leg being trained during flow-restriction induced by lower body positive pressure. The EPR was assessed by measuring the increase in heart rate (HR) and mean arterial pressure (MAP) during an isometric knee extension of 35% of max torque for 90 s, this was done before (C), and after training in each leg (R and NR, respectively).

**Results:**

At the end of isometric contraction, the increase in mean AP (MAP) in the NR-trained leg and in the control condition were 41 ± 4 and 38 ± 4 mmHg, respectively, whereas the increase in the R-trained leg was 30 ± 4 mmHg (*p* < 0.05 R vs C and NR), corresponding to a decrease of about 25%. A similar patter was observed with respect to responses in HR, where the increase was 28 ± 3 and 28 ± 3 bpm in the NR and C, and 22 ± 4 in the R condition (*p* < 0.05 R vs C and NR).

**Conclusions:**

Peripheral metabolic changes induced by relative ischemia are important in modifying the EPR in response to exercise training.

## Introduction

It is generally admitted that the cardiovascular response to physical exercise implies the concerted action of a central command mechanism, and of the exercise pressor reflex (EPR), with influence from the cardiopulmonary reflex (Rowell and O’Leary 1990; Michelini et al. 2015; Fadel and Raven 2012). As hypothesized, the EPR implies mechano- and chemo-sensitive receptors that affect afferent nerve traffic in c-fibers, which in turn increase the sympathetic outflow to the heart and resistance vessels (Rowell and O’Leary 1990; Seals et al. 1988). This is possibly accompanied by a postulated simultaneous withdrawal of vagal tone to the heart, which occurs in the first seconds of exercise (Fagraeus and Linnarsson 1976; Lador et al. 2008). The ensemble of these responses lead to increased heart rate (HR) and arterial pressure (AP), and is important for a proper hemodynamic response during physical exercise in healthy humans (Amann et al. 2011). The relative role of these mechanisms in generating the cardiovascular response to exercise is still matter of debate. Insight on the role of the EPR may come from endurance training, as long as it was previously shown that endurance training blunts the exercise pressor reflex (EPR) (Somers et al. 1992; Mostoufi-Moab et al. 1998; Ray 1999) and that endurance-trained subjects have a lower EPR than untrained and strength-trained subjects (Kolegard et al. 2013).

The aim of the present study was to investigate whether aerobic training with and without relative ischemia would act to modify the EPR. The experimental model used was single leg exercise in which one leg exercise was done with restricted blood flow, inducing a higher metabolic stress and activation of the muscle chemoreflex (Eiken and Bjurstedt 1987), and the other leg being trained without any blood flow restriction but with identical workload.

Our main hypothesis was that flow restriction during training at a given workload (R-training), would act to decrease the EPR when comparing with regular training on the same workload (NR-training). It was further hypothesized that the difference in EPR would be shown towards the end of the isometric contraction, when the accumulation of metabolites and fatigue occurs. A significant inter-individual variability was expected with regards to the training effect on peak performance.

## Methods

Ten healthy male subjects participated in the study. Their ages, weights, heights and VO_2_-max were [mean (range)] 24 (20–27) years, 78 (68–95) kg, 181 (173–191) cm, and 50 (45–57) ml/min/kg. Because of the strenuous nature of the training program, some familiarity with physical training was considered important, whereas recruiting subjects that were too well-trained (> 60 ml/min/kg) was avoided, as this might diminish the possibility of detecting significant training effects. The subjects were informed about the experimental procedures and the nature of the training program before consenting to participate. The study was performed in accordance with the Declaration of Helsinki and the ethics committee at Karolinska Institutet, Stockholm, approved the protocol and experimental procedures. Results from this study, related to gene analysis and skeletal muscle tissue adaptations, have been used in other publications (Rullman et al. 2009; Gustafsson et al. 2007).

### Exercise model

A method first described by Eiken and Bjurstedt (1987) was used for induction of restricted blood flow during exercise. Local application of increased pressure around the working leg was used to reduce exercise blood flow in a controlled fashion. The subject was positioned supine in the opening of a pressure chamber with the upper body outside and both legs inside the chamber and one leg strapped to a pad on the lower leg. The dynamic constant-load one-legged knee-extension exercise was done in a similar fashion as earlier described (Andersen et al. 1985). Each voluntary contraction extended the leg from 70° to 150° knee angle. Flexion was performed passively using the ergometer flywheel momentum to move the leg for the next extension. The chamber opening was sealed off at the level of the crotch by a rubber diaphragm with holes and self-sealing sleeves for the legs. Shoulder supports were used to prevent displacement of the body as the chamber pressure was increased. For exercise under restricted blood flow, the chamber pressure acting on the working leg was elevated to 50 mm Hg above atmospheric pressure. This has been shown to reduce leg blood flow during one-legged cycle exercise by 15–20%, reduce 10–12% units of venous oxygen saturation and a greater depletion of ATP in the working muscle and release of lactate (Sundberg 1994). Exercise under non-restricted blood flow condition was done using the same experimental arrangements but at current atmospheric pressure.

### Training protocol

One-leg training was performed four times a week for 5-weeks, giving a total of 20 training sessions. One leg trained under restricted blood flow condition (R-leg), providing relative ischemia during exercise, while the other leg trained with non-restricted blood flow (NR-leg). The subjects were randomized into two groups; one group trained their right leg and the other group their left leg during restricted blood flow. Each training session began by 45 min of training under restricted blood flow condition. The subjects were instructed to perform knee extensions at a constant rate of 60/min at the highest tolerable workload for 45 min, taking in to account that the whole 45-min session must be accomplished. The workload was changing during the training session; hence the load was adjusted to maintain high intensity while still ensuring that the bout could be completed without interruptions. After 10 min of rest, the same workload protocol was performed by the other leg, but with normal leg blood flow. Accordingly, the two legs developed the same power and amount of work in each session. The subjects were encouraged to achieve a subjective rating of perceived leg effort (Borg scale 6–20) during training with flow restriction above 15 (hard) for the last 5 min in all training sessions. The Borg scale was designed for rating of perceived exertion during general exercise with large muscle groups, but has also been validated for use in exercise with smaller muscle groups (Garcin et al. 1998). The R-leg training was invariably experienced as extremely strenuous with periods of ischemic muscle pain occurring frequently during the training session, whereas training of the NR-leg was experienced as very light. The cumulative (total) work load per bout (W*min) was calculated (Table 1).

**Table 1:** Training intensity, perceived exertion and heart rate during training

Total work load, first training bout (W × min)	503 ± 181
Total work load, last (20th) training bout (W × min)	848 ± 100^a^
Perceived exertion (units), R-training	17 (15–18)
Perceived exertion (units), NR-training	12 (9–13)^b^
Heart rate (bpm), R-training	98 ± 11
Heart rate (bpm), NR-training	89 ± 7^b^

Mean ± SD, Median (range)

^a^Significant difference between first and last exercise bout

^b^Significant difference compared to R-trained leg. Perceived exertion and heart rate were recorded at the first and last exercise session and the presented values denotes the average in the R and NR condition, respectively

### Performance test

After familiarization with the experimental model, one-legged step-wise incremental exercise tests were performed during the week before the training period and the week after the training period. Each leg was tested during non-restricted blood flow conditions. The subject was instructed to maintain 60 knee extensions per min, starting with 2 min at 5 W. The workload was then increased by 5 W every min up to 20 W and then by 2.5 W until the kicking rate could no longer be maintained. When the rate fell below 55 rpm for more than 5 s, time and peak load were recorded and the experiment terminated.

### Exercise pressor response test

Isometric torque (maximal voluntary contraction; MVC) was determined during knee-extension contractions in both legs using a dynamometer (Cybex II, Lumex Inc. N.Y., USA). During the knee extension, the subject sat in a chair with a vertical back support. The thighs and pelvis were secured with Velcro straps. The dynamometer lever arm was attached proximal to the ankle with the knee joint at an angle of 90°. The subject was instructed to produce maximal force as rapidly as possible without kicking, and to sustain maximal force for 5 s. The procedure was repeated three times with a 30-s rest period between contractions. Torque was recorded with a computer-based data acquisition and analysis system (BioPac Systems, Santa Barbara, USA). Using the same experimental arrangements as for the MVC measurements, the subject performed a 90-s sustained isometric knee-extensor contraction at 35% MVC. The subject was given visual feedback from a torque-gauge display to keep the pre-set torque level. To avoid straining (Valsalva) maneuvers during the contractions, the subject was also instructed to continuously count out loud during the contractions. The procedure was done once in each leg before and after the 5-week training period. The 35% MVC defined before the training period was used also after training, e.g., the same absolute torque during isometric contraction before and after. Heart rate (HR) and arterial pressure (AP) were measured beat-by-beat with a volume clamp technique (Finapres 2300, Ohmeda, Englewood, OH, USA) with a cuff placed around the mid phalanx of the third finger of the right arm. The arm was supported by a mitella and the finger was kept at heart level. The Finapres method is widely used and known to reliably follow changes in MAP in various conditions, the absolute values might differ compared to invasive measurements (Azabji Kenfack et al. 2004), but these changes are typically systematic. ECG was recorded with the electrodes positioned in a 5-lead precordial arrangement using a cardiomonitor (Physio-Control Lifepak 8, Cardiomonitor, Physio-Control Corp, Redmond, USA). AP, ECG, and torque were recorded with the same acquisition systems as mentioned above. Offline mean AP (MAP) was computed as the arithmetic mean between systolic peaks and stored as a level during that interval. The cardiovascular response to the isometric contraction was characterized by measurements of the increase in HR and MAP at four different times during the isometric contraction. HR and MAP were averaged over 5 s at 20, 40, 60, and 80 s after the initiation of contraction. These measurements were done on both legs before and after the training period. The responses from both legs before training were averaged in each subject and this average was used as the control when comparing to the responses after R- and NR-training respectively.

### Statistics

The difference in the cardiovascular exercise response before and after training with and without flow restriction was tested using a 2-way ANOVA with repeated measurements. If the ANOVA indicated a significant difference (*p* < 0.05), a post hoc analysis, planned comparison, was used to locate at what times during the isometric contraction that the difference occurred. 2-way ANOVA was also used for testing the difference in the peak load test and maximum isometric torque before and after training. Student’s *t* test was used to analyze the change in training load early and late during the training period, and also for testing the difference in perceived exertion and HR during R- and NR-training respectively.

## Results

All 10 subjects successfully completed the 20 exercise sessions and the related experiment procedures. The average total workload during an exercise session increased from 503 ± 181 W × min at the first exercise bout to 848 ± 100 W × min during the last bout (*p* < 0.01) (Table 1). Overall, the perceived exertion and heart rate (HR) were higher during R-training vs NR-training (Table 1).

### Performance tests

**Fig. 1:**
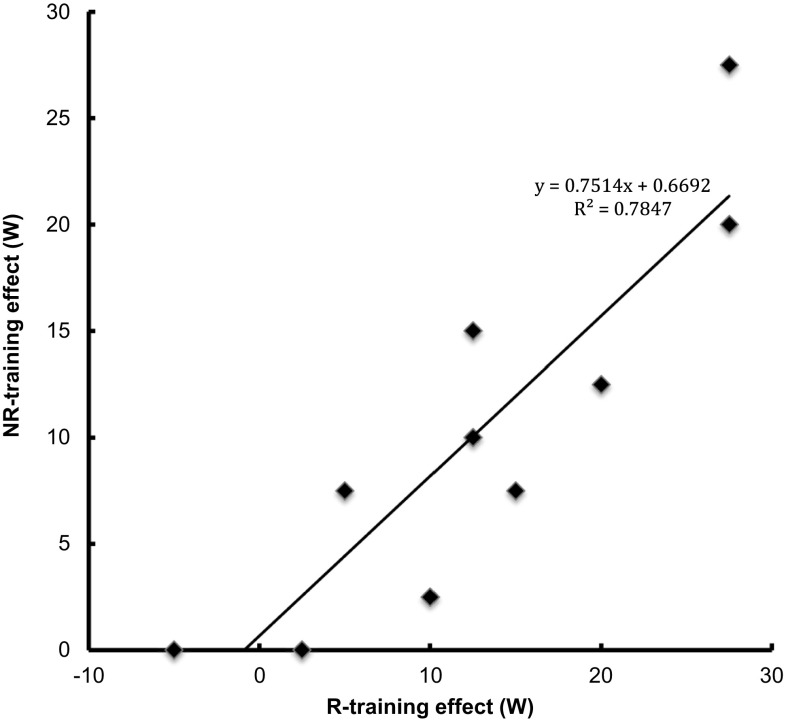
Change in the peak work load during the performance test after 5 weeks of training, in the R- and NR-trained leg of each subject

Peak load at the performance test increased after both R and NR training, from 43 ± 3 to 56 ± 2 W and from 41 ± 3 to 51 ± 2 W, respectively (main effect *p* < 0.05). There were no significant differences between the R- and NR-trained legs (Table 2). There was a large inter-individual variability regarding the change in peak performance, where subjects with larger training effect in the R-trained leg also had larger training response in the NR-trained leg (Fig. 1). Isometric knee-extension torque increased after training in both the R- and NR-trained legs, from 278 ± 20 to 283 ± 22 Nm and from 279 ± 17 vs 293 ± 20 N m, respectively (main effect *p* < 0.05), whereas no significant difference was noted between the R- and NR-trained leg (Table 2).

**Table 2:** Peak load test and maximum isometric torque

	R-trained leg	NR-trained leg
Peak load, control (W)	43 ± 3	41 ± 3
Peak load, after training (W)	56 ± 2^a^	51 ± 2^a^
Isometric torque, control (Nm)	278 ± 20	279 ± 17
Isometric torque, after training (Nm)	283 ± 22^a^	293 ± 20^a^

Mean ± SE

^a^Significant difference compared to control in the same leg (*p* < 0.05), main effect of training, no difference between, R- and NR-trained leg

### Exercise pressor test

**Fig. 2:**
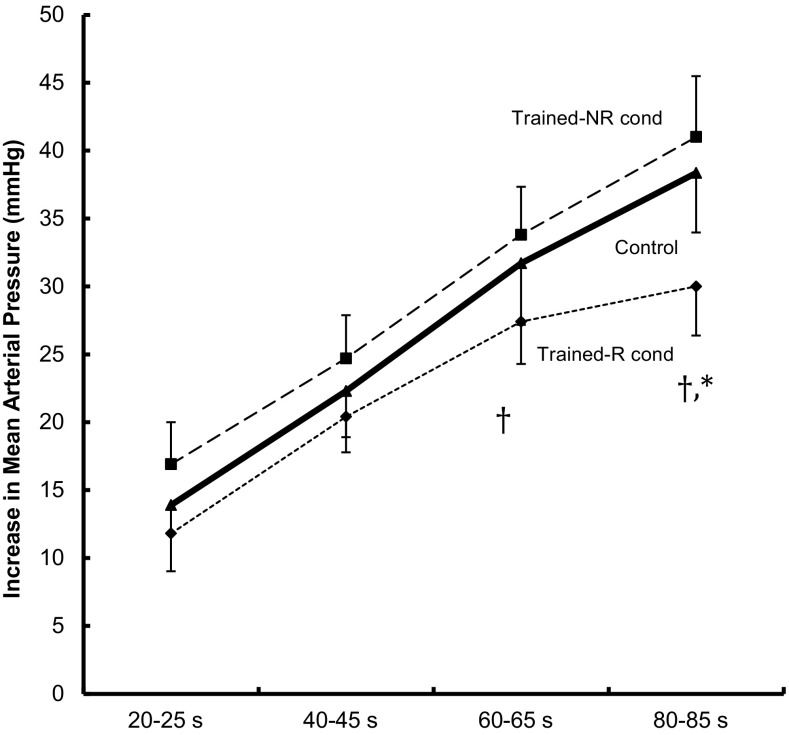
Mean arterial pressure responses to 90 s isometric unilateral knee extension at 35% of maximal voluntary contraction. Control; mean response from both legs before training, Trained-NR cond; response while contracting the leg that had received non-ischemic training, and Trained-R cond; response in the leg that had undergone ischemic training. *n* = 10, values are mean ± SE. ^†^Significant difference between NR and R condition at a given time interval, *significant difference between control and R condition at a given time interval (*p* < 0.05)

**Fig. 3:**
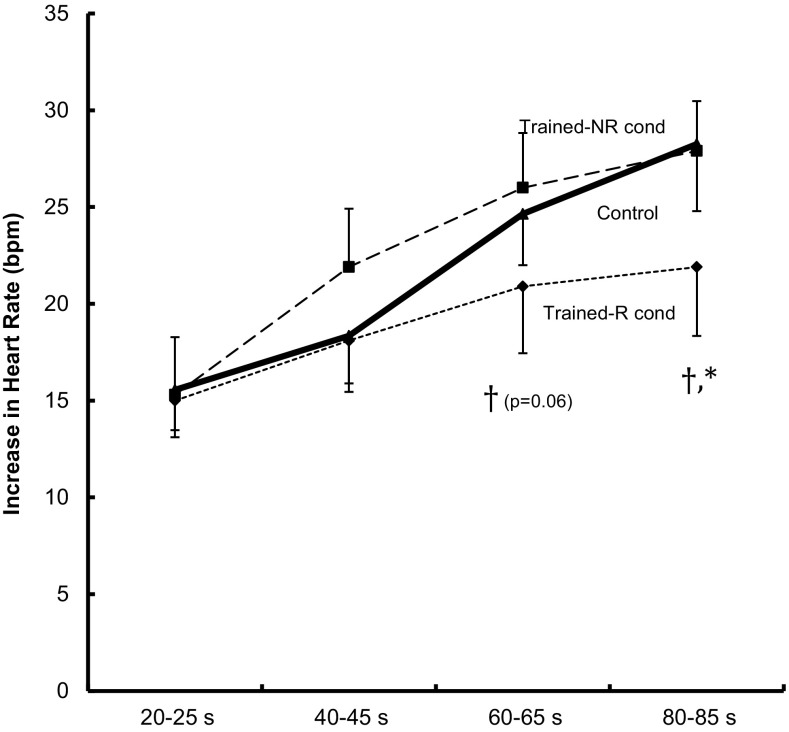
Heart rate responses to 90 s isometric unilateral knee extension at 35% of maximal voluntary contraction. Control; mean response from both legs before training, Trained-NR cond; response while contracting the leg that had received non-ischemic training, and Trained-R cond; response in the leg that had undergone ischemic training. *n* = 10, values are mean ± SE. ^†^Significant difference between NR and R condition at a given time interval, *significant difference between Control and R condition at a given time interval (*p* < 0.05)

Figures 2 and 3 show mean responses to 90 s isometric knee extensions at 35% of MVC, before training (control) and after training with and without ischemia, N and NR condition, respectively. Absolute values of MAP and HR are denoted in Table 3. There was in general a similar increase up to 45 s in all conditions, both in MAP and HR responses. After 60 s, the pressor response in the R-leg diverged from the control and the NR-leg, displaying less increase in both MAP and HR. There was no significant difference between control and NR condition at any time point.

**Table 3:** Mean arterial pressure (MAP) and heart rate (HR) before and after EPR

	Control	NR-trained leg	R-trained leg
MAP (mmHg)			
Baseline	94 ± 7	97 ± 7	96 ± 8
End EPR	133 ± 17	138 ± 17	126 ± 13
HR (bpm)			
Baseline	78 ± 11	80 ± 12	80 ± 12
End EPR	106 ± 12	108 ± 15	101 ± 17

Mean ± SD

Hence after 80–85 s of isometric contraction, the increase in MAP in response to isometric contraction in the NR-leg and in the control condition were 41 ± 4 and 38 ± 4 mmHg, respectively, whereas the increase in the R leg was 30 ± 4 mmHg, corresponding to a decrease of about 25%. A similar patter was observed with respect to response in HR at the last time interval, where the increase was 28 ± 3 and 28 ± 3 bpm in the NR and control condition, and 22 ± 4 in the R condition. The response at 80–85 s in the R condition was significantly lower than the responses in the NR and control conditions, both for MAP and HR (*p* > 0.05). At the 60–65 s period, the R condition was significantly different to the NR condition but not to the control condition, both concerning the responses in MAP and HR.

## Discussion

This study indicates that relative ischemia induced by flow restriction during exercise appear to decrease the exercise pressor reflex. Furthermore, the fact that the difference in responses occurred only during the second half of an isometric contraction suggest that the altered exercise pressor response depends on an altered afferent signaling originating from the muscle chemoreflex or a blunted increase in central command due decreased muscle fatigue.

It has previously been shown that endurance training of the forearm reduces the increase in muscle sympathetic nerve activity (MSNA), but not MAP or HR, during isometric handgrip (Somers et al. 1992). Mostoufi-Moab et al. (1998) indicated that endurance training of the forearm, in this case reduced the increase in MAP during flow-restricted dynamic exercise, and also decreased lactate accumulation and blunted the decrease in pH. A similar adaptation was demonstrated during one-legged training, where MSNA and MAP were decreased during dynamic knee extension of 40 W after 6 weeks of training (Ray 1999). In the present study, both legs were trained with the same workload, with one leg being under stronger metabolic stress i.e. with increased lactate release and reduced pH due to an imposed restriction of blood flow (Sundberg and Kaijser 1992). The advantage of such a model is exclusion of inter-individual genetical differences in the comparison of the two conditions and the utilization of small muscle mass with minor hemodynamic effects makes it possible to relates any obtained findings to the peripheral tissue. The present results indicate that the metabolic disturbance *per se* induces peripheral adaptations that reduce the EPR. There were no observed changes in EPR after NR-training, despite about 20% increase in peak performance (Table 2). This could be coupled to the relative low intensity and slight metabolic disturbance in this condition compared to R-training as also reflected in the perceived exertion (Table 1). An increased intensity during NR-training might have had an effect on the EPR; however, the current findings indicate that any such effect would likely then be coupled to relative hypoxia with partly anaerobic metabolism.

The difference between the R- and NR-trained leg, in both the HR and MAP exercise pressure responses, occur in the 2nd half of the isometric contraction (Figs. 2, 3). The cardiovascular response of an isometric contraction comprises of effects from central command, peripheral chemo- and mechano-reflexes (Rowell and O’Leary 1990). The chemoreflex comes into effect after some time, because of its dependence of the gradual accumulation of metabolites and decrease in pH (Victor et al. 1988; Seals et al. 1988; Pryor et al. 1990). Hence, one conclusion could be that R-training primarily alters the chemoreflex, either by less accumulation of substances, due to an augmented muscle adaptation, that triggers the reflex or by a blunted afferent signal. An alternative mechanism, partly related to reduced accumulation of metabolites, could be that R-training decreases the muscle fatigue (Eiken et al. 1991) in a way that acts to damp the increase in central command over time during the isometric contraction (Fisher and White 1999; Schibye et al. 1981).

Regardless of mechanism, it has been shown that fibers with aerobic rather than glycolytic profile, have a smaller exercise pressor response (Petrofsky and Lind 1980; Wilson et al. 1995) and a better endurance during isometric contractions (Hulten et al. 1975). Previous studies have indeed shown that R-training, performed as in the present study, increases the aerobic capacity (Kaijser et al. 1990; Gustafsson et al. 2007; Esbjornsson et al. 1993), and acts to alter the metabolic capacity towards a more aerobic profile with an increased capillary density (Eiken et al. 1991; Esbjornsson et al. 1993). In line with this reasoning, it has also been shown that endurance-trained individuals have a lower exercise pressor response during isometric contraction of the quadriceps muscle, compared to untrained subjects (Kolegard et al. 2013). In addition to the effects of differences in metabolic pathways on metabolic end products there are several additional mechanisms that might affect receptors coupled to group III and IV muscle afferent fibers differently (Kaufman and Forster 1996). In fact, receptor density might differ between types of muscle, buffering capacity has been shown to be different (Sahlin and Henriksson 1984), and afferent neurons in oxidative fibers appear to respond different compared to those in more glycolytic fibers (Xing et al. 2008).

There was large variation between subjects related to the magnitude of the training effect (Fig. 1), which is commonly seen in training studies (Lortie et al. 1984; Prud’homme et al. 1984; Bouchard et al. 2011). The inherent nature of adaptive responses to physical exercise was further supported by the observation that subjects with large training effects in the NR-trained leg also had large effects in the R-trained leg (Fig. 1).

### Methodological consideration

The EPR test done before and after training was performed with the same absolute power defined as 35% of maximal knee torque (MVC) assessed before training. Hence since the MVC increased after training (Table 2), with approximately 2% in the R-trained leg and 5% in the NR-trained leg, it could be argued this load corresponds to less than 35% and thus might explain a lower magnitude of the EPR. However, since a decrease in EPR was only apparent in the R-leg, to an extent that provides significant difference compared to the NR condition, despite a numerically larger increase in MVC in the NR condition, any such effect would have no effect on the current conclusions.

## Conclusions

The present study indicates that the ischemic component during exercise changes the EPR during isometric exercise.

The differences in EPR appear towards the end of the contraction indicating an effect of an altered muscle chemoreflex response and/or decreased muscle fatigue coupled to a decrease in central command. Understanding the plasticity of the exercise pressor reflex has relevance not only for healthy individuals, but also for patient groups such as heart failure patients if this reflex is exaggerated and acts to decrease exercise capacity (Amann et al. 2014).

## Abbreviations

ANOVA: Analysis of variance
AP: Arterial pressure
C: Control
EPR: Exercise pressor reflex
HR: Heart rate
MAP: Mean arterial pressure
MSNA: Muscle sympathetic nerve activity
MVC: Maximal voluntary contraction
NR: Non flow-restricted
R: Flow-restricted
